# Primary vitreoretinal lymphoma: diagnostic and therapeutic insights from a Slovenian population-based study

**DOI:** 10.1186/s40942-026-00830-5

**Published:** 2026-03-13

**Authors:** Nika Vrabič, Lučka Boltežar, Matej Panjan, Veronika Kloboves Prevodnik, Polona Jaki Mekjavič, Marija Skoblar Vidmar, Pia Klobučar, Mojca Globočnik Petrovič, Nataša Vidovič Valentinčič

**Affiliations:** 1 Faculty of Medicine, University of Ljubljana, Vrazov trg 2, Ljubljana, Slovenia; 2 Eye Hospital, University Medical Centre Ljubljana, Grablovičeva ulica 46, Ljubljana, Slovenia; 3 Institute of Oncology Ljubljana, Zaloska cesta 2, Ljubljana, Slovenia; 4 Faculty of Medicine, University of Maribor, Taborska 2, Maribor, Slovenia

**Keywords:** Primary vitreoretinal lymphoma, Population-based study, Methotrexate, Radiotherapy, Survival analysis

## Abstract

**Background:**

This study aimed to determine the national incidence, characterize clinical features, and evaluate the treatment outcomes of primary vitreoretinal lymphoma (PVRL) in Slovenia.

**Methods:**

We conducted a population-based, retrospective case series of all patients diagnosed with PVRL at the Eye Hospital, University Medical Center Ljubljana, between January 2013 and May 2024. The diagnosis was histopathologically confirmed, and primary central nervous system involvement was excluded. Clinical presentation, diagnostic delay, treatment modality, and adverse events were analyzed. Progression-free survival (PFS), overall survival (OS), and lymphoma-specific survival (LSS) were calculated.

**Results:**

Twelve patients were diagnosed with PVRL (four men, eight women; median age of 76 years). The average annual incidence was 0.52 cases per million. The median time from symptom onset to diagnosis was 238 days. The two most common symptoms were decreased visual acuity (75%) and floaters (58%). Vitreous cellular infiltration was the predominant clinical sign and was observed in 92% of patients. Five patients presented with unilateral disease, seven with bilateral disease, and three with unilateral disease that progressed to bilateral involvement. The median follow-up was 31.5 months. Eleven patients received one or more treatment modalities: intravitreal rituximab and/or methotrexate, local radiotherapy, and/or systemic chemotherapy. Local remission was achieved in all treated eyes. Two cases of granulomatous uveitis occurred during intravitreal rituximab therapy. The median PFS was 12 months; the two- and three-year PFS rates were 37.5% and 18.8%, respectively. The median OS was not reached; the two- and three-year OS rates were 70% and 56%, respectively. The LSS was 80% at two years and 64% at three years.

**Conclusions:**

This Slovenian population-based study provides real-world insights into PVRL management. In elderly and medically fragile patients, local treatment modalities provided effective ocular disease control with acceptable toxicity.

## Background

Primary vitreoretinal lymphoma (PVRL) is a rare intraocular malignancy classified as diffuse large B-cell lymphoma and a subtype of central nervous system (CNS) lymphoma that primarily affects retinal and vitreal tissue [1]. Its estimated incidence is 0.047 cases per 100,000 annually, reflecting a twofold increase since 1990, which has been attributed to factors such as population aging, advances in diagnostic methods, and greater clinical awareness [2]. Primary CNS lymphoma accounts for 4–6% of all brain tumors and fewer than 1% of non-Hodgkin lymphomas [3], typically affecting adults between their fifth and ninth decades of life [4–6].

Diagnosing PVRL is challenging due to its variable clinical presentation, often leading to diagnostic delays of several months or even years. Vitreoretinal biopsy remains the diagnostic gold standard, and CNS and systemic involvement must be ruled out to confirm disease localization.

The rarity of PVRL and limited clinical research have hindered the establishment of standardized treatment strategies. According to recent European Hematology Association and European Society for Medical Oncology (EHA–ESMO) guidelines, high-dose methotrexate-based regimens are recommended as frontline therapy, with intravitreal methotrexate for the rapid control of intraocular disease [7]. Nonetheless, treatment often requires individualized approaches due to the rarity of the disease and the complexity of its management.

The present study aims to determine the national incidence, clinical characteristics, and treatment outcomes of PVRL in Slovenia. The analysis is population-based since the participating centers constitute the only institutions nationwide that provide care for patients with PVRL.

## Methods

### Patients

This population-based, single-center, retrospective case series comprehensively analyzed the clinical data of all patients diagnosed with PVRL at the Eye Hospital, University Medical Center (UMC) Ljubljana, the only Slovenian referral center for PVRL, between January 2013 and May 2024.

Complete national case ascertainment verified through: sole PVRL referral center status, cross-check with National Cancer Registry, and no competing diagnostic/treatment centers.

The diagnosis was confirmed via intraocular tissue biopsy. To exclude primary CNS involvement, all patients underwent systemic imaging and systemic staging to exclude other primary lymphoma sites. Each case was reviewed by a multidisciplinary lymphoma tumor board, and treatment was delivered according to the therapeutic modality: radiotherapy and systemic therapy at the Institute of Oncology Ljubljana and intravitreal therapy at the Eye Hospital, University Medical Center Ljubljana. Follow-up data for the specified period were retrieved from the electronic health records of the Eye Hospital UMC and the Institute of Oncology, Ljubljana.

All patients provided written informed consent to participate in the study, which was conducted in accordance with the principles of the Declaration of Helsinki.

### Predefined study variables of interest

The following variables were explored: time to diagnosis, defined as the interval from symptom onset to definitive histopathological confirmation; clinical presentation, including initial symptoms and ophthalmic findings; laterality of disease at diagnosis and during follow-up; treatment modalities categorized by intravitreal therapy, radiotherapy, or systemic chemotherapy; treatment-related toxicity documented as adverse events requiring therapy modification or discontinuation; and visual function assessed via best-corrected visual acuity (BCVA) at baseline and final follow-up.

We further explored: progression-free survival (PFS), defined as the time from the end of systemic treatment, intravitreal therapy or radiotherapy to disease progression, including contralateral eye involvement, CNS progression, systemic progression, or death from any cause; overall survival (OS), defined as the time from diagnosis to death from any cause; and lymphoma-specific survival (LSS), defined as the time from diagnosis to death directly resulting from lymphoma.

Exploratory analyses evaluated potential associations between survival outcomes (PFS, OS) and clinical or treatment factors, including the bilateral nature of the disease, treatment modality, time to diagnosis, disease progression, and patient age. Survival outcomes and prognostic trends were systematically compared to published literature, particularly meta-analyses reporting median survival.

### Clinical ophthalmic examination

All patients underwent a comprehensive ophthalmic examination, including BCVA testing, tonometry, slit-lamp biomicroscopy, and dilated fundus examination. Clinical data were recorded at baseline and at the pre- and posttreatment follow-up visits. Vitreous cellular infiltration was quantified according to the Standardization of Uveitis Nomenclature criteria [8]. BCVA was reported in Snellen decimal values and converted to logMAR values. Counting finger vision was documented in logMAR 2, hand motion in 3, and light perception and no light perception were not converted to logMAR [9].

### Staging

Initial imaging, magnetic resonance imaging (MRI) of the head, and positron emission tomography (PET) scans, was reviewed to exclude primary CNS lymphoma or systemic involvement. Contrast-enhanced brain MRI (gadolinium) was performed using the following sequences: T1-weighted (pre- and post-gadolinium), T2-weighted, fluid-attenuated inversion recovery (FLAIR), and diffusion-weighted imaging (DWI). In one patient, baseline CNS imaging documentation was incomplete; although follow-up clinical data were consistent with isolated ocular disease, occult CNS involvement at presentation cannot be entirely excluded. Computed tomography was performed in another patient due to the presence of an MRI-incompatible implant. Lumbar puncture was performed selectively in patients with strong clinical suspicion of CNS involvement (neurological symptoms, aggressive ocular disease, systemic abnormalities) or when CSF analysis was needed for diagnostic confirmation. Follow-up imaging findings were analyzed, with particular attention given to the location and timing of any extraocular spread of PVRL.

### Diagnostics

This long-term study (2013–2024) reflects diagnostic standards of the period. Molecular testing for MYD88 L265P mutation and IL-6/IL-10 levels was not performed due to a lack of local laboratory validation and availability during most of the study period. Diagnosis relied on cytopathologic examination, flow cytometry, and repeated sampling when initial results were inconclusive. Data on the initial clinical diagnosis and diagnostic workup were collected. Detailed cytologic and silicone fine-needle aspiration biopsy (s-FNAB) [10] results were obtained. Intraocular samples were sent to the Department of Cytopathology at the Institute of Oncology, Ljubljana. The samples were processed according to the standard protocols routinely used at the Institute of Oncology Ljubljana.

Specifically, Giemsa- and Papanicolaou-stained cytospins were prepared from each sample for microscopic evaluation. The remaining sample material was used for flow cytometric immunophenotyping, following the protocol previously described by our group [11]. Notably, monoclonal antibodies against CD45, CD19, CD20, CD3, CD56, CD10, CD5, kappa, lambda, CD38, CD14, TCR gamma/delta, CD4 and CD8 (all BD Biosciences) were used. After a 20-minute incubation with the antibodies, the samples were measured via either a six-color flow cytometer (FACSCanto II, BD Biosciences) or a ten-color flow cytometer (FACSCanto 10, BD Biosciences). The data were analyzed via CellQuest (BD Biosciences) for FACSCanto II or BD FACSDiva software (BD Biosciences) for FACSCanto 10. If needed, additional molecular studies were performed at the Department of Molecular Diagnostics, Institute of Oncology Ljubljana. Molecular testing for MYD88 L265P mutation and IL-6/IL-10 levels was not performed due to a lack of local laboratory validation and availability during most of the study period. Diagnosis relied on cytopathologic examination, flow cytometry, and repeated sampling when initial results were inconclusive.

### Therapy

Data on local therapies, including intravitreal treatment with rituximab or methotrexate and ocular, orbital, and whole-brain irradiation, were collected, and associated adverse effects were recorded. Systemic chemotherapy regimens and radiation doses were documented, and irradiation techniques and dosimetry were analyzed.

Remission was defined as the absence of active ocular lymphoma, as evidenced by a clear vitreous and resolution of prior retinal or optic nerve infiltrates if present [12]. For the intravitreal treatment, a protocol consisting of an induction phase of two weekly injections for 1 month, followed by a consolidation phase of weekly injections for 2 months, and a maintenance phase of monthly injections for 9 months was used [13].

Patients were censored at the last recorded visit. Survival status was verified through the National Cancer Registry as of 5 September 2025.

### Statistical methods

For demographic data, the median values, along with interval ranges, were reported. Survival data were analyzed with SPSS Statistics, version 26 (IBM, Armonk, NY, USA).

## Results

### Demographic data

The cohort included 12 patients (four men and eight women, male-to-female ratio of 1/2.25). The median age at symptom onset was 75 years (range, 49–91 years), whereas the median age at diagnosis was 76 years (range, 51–91 years) (Table 1). Annually, 0–3 patients were diagnosed with PVRL, with an average yearly incidence of 0.52 cases per million from January 2013 to January 2024.

### Time to diagnosis and clinical characteristics

The median time from symptom onset to diagnosis was 238 days (range, 51 days to 3 years and 3 months). Eleven patients reported no known immunodeficiency, whereas one patient was receiving systemic methotrexate and etanercept for rheumatoid arthritis at the time of PVRL diagnosis. No patient had a prior history of extraocular lymphoma. Imaging studies, including CT, PET, or MRI, were performed in all patients to exclude primary CNS involvement, and LP was performed when feasible. Whole-body PET scanning was a key component of the diagnostic evaluation in nine patients.

**Table 1:**
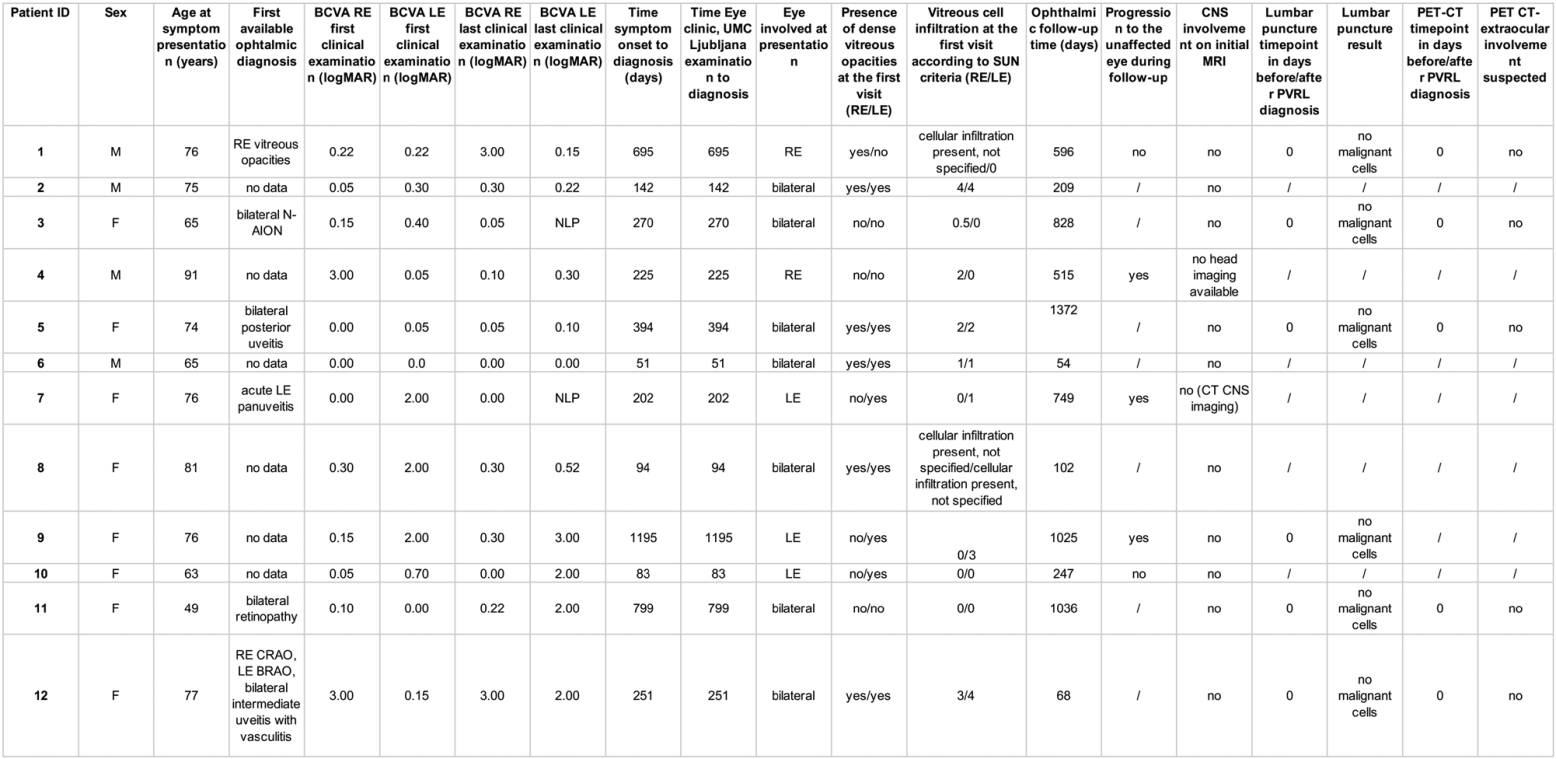
Demographic data

BCVA – best-corrected visual acuity, RE – right eye, LE – left eye, CNS – central nervous system, PET-CT – positron emission tomography–computed tomography, N-AION - nonarteritic anterior ischemic optic neuropathy, NLP – no light perception, BRAO - branch retinal artery occlusion, CRAO - central retinal artery occlusion

### Clinical presentation

#### Reported symptoms and clinical findings

The most commonly reported symptoms at presentation included a subjective decrease in visual acuity or blurred vision in nine patients, floaters in seven patients, central scotomas in two patients, flashes of light in two patients, eye pain due to increased intraocular pressure in one patient, altered color vision in one patient, and photophobia in one patient.

The referral ophthalmic diagnoses available for six patients included vitreous opacities, nonarteritic anterior ischemic optic neuropathy, panuveitis, posterior uveitis, retinopathy, and retinal artery occlusion associated with intermediate uveitis and retinal vasculitis (Table 1).

#### Ophthalmic examination

At the first slit-lamp examination at our institution, vitreous cell infiltration was observed in 11 of the 12 patients (91.7%), representing the most frequent and consistent clinical finding. The remaining patient presented with dense vitreous opacities. In several cases, vitreous haze was severe enough to obscure adequate visualization of the fundus, limiting the documentation of retinal findings. Retinal involvement was therefore not analyzed because of incomplete and inconsistent clinical records. Optic nerve infiltration was identified in one patient.

Baseline BCVA was documented for all patients, with BCVA in the affected eyes ranging from 0.00 to 3.0 logMAR and 0.00 to no light perception at the last follow-up.

#### Follow-up

All patients had at least two visits to the Eye Hospital, UMC Ljubljana. The median follow-up from diagnosis was 31.5 months.

### Results of vitreous and retinal biopsies and cytopathological analyses

**Fig. 1:**
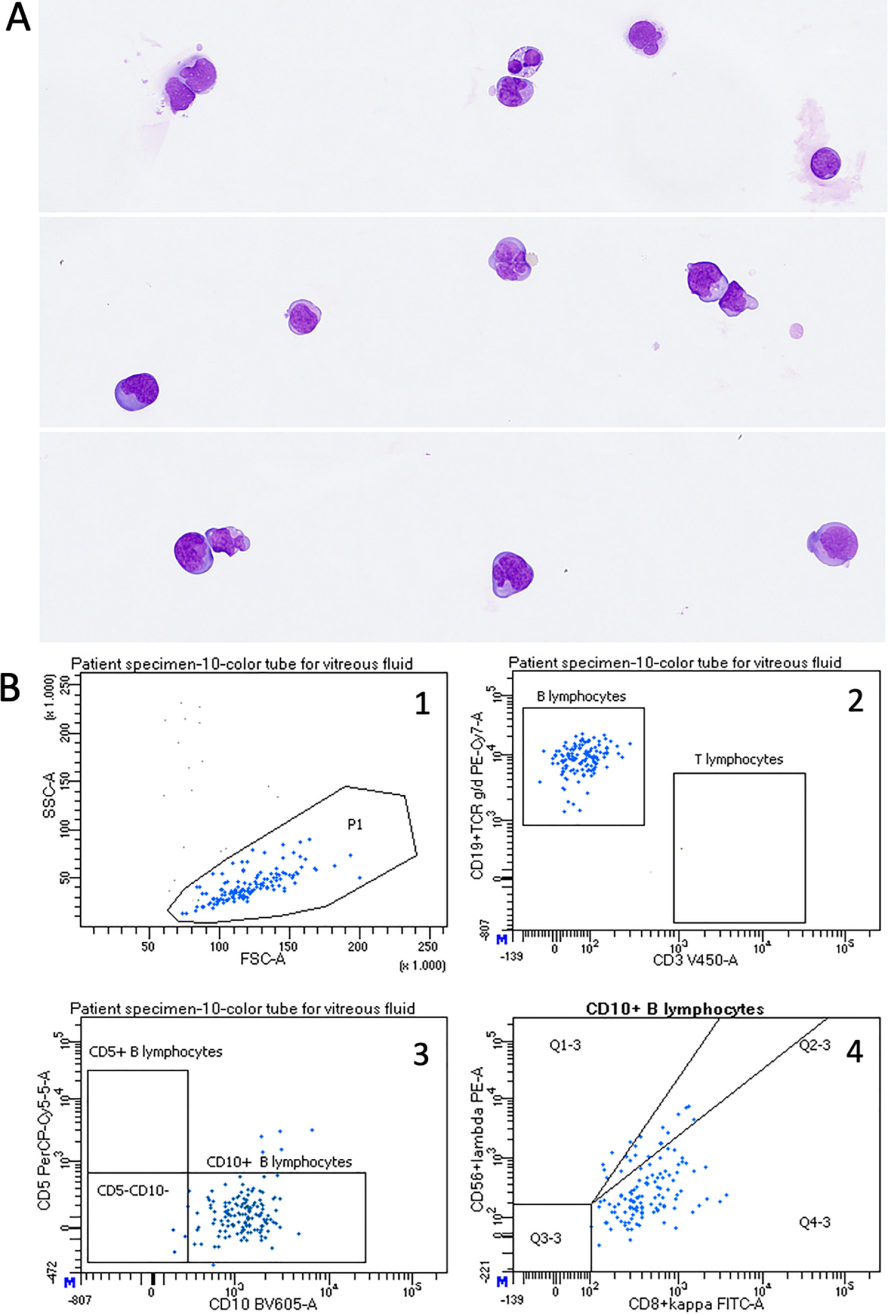
Microscopic and flow cytometric results in a vitreous sample from a patient with primary intraocular lymphoma. **A**) Gallery of lymphoma cells generated from digital images of Giemsa stained cytospin. **B**) Flow cytometric results demonstrating a population of large lymphoma cells (dot plot 1: FCS/SCS) expressing CD19 (dot plot 2: CD19/CD3), CD10 (dot plot 3: CD10/CD5), and surface kappa light chains (dot plot: 4: kappa /lambda)

PVRL, classified as diffuse large B-cell lymphoma, was confirmed by intraocular vitreous and/or retinal biopsy in all patients. Vitreous biopsy was performed in all patients, and retinal biopsy was additionally performed in five patients. Retinal biopsy was performed via full-thickness transvitreal tissue chorioretinal biopsy in one patient and via S-FNAB in four patients, as previously described [10]. Flow cytometric analysis was performed in all cases and supported the cytopathological diagnosis of PVRL in 10/12 (83.3%) cases (Fig. 1). In addition, gene rearrangement studies using BIOMED 2 method confirmed clonality of lymphoma cells in 5/7 (71.1%) cases, while MYD88 mutation was detected in 3/4 cases. Notably, in one case with negative by flow cytometric findings, detection of a MYD88 mutation enabled the diagnosis of PVRL.

### Treatment strategies

A multidisciplinary tumor board, including an ophthalmologist, a medical oncologist, and a radiation oncologist, was convened to individualize treatment plans for each patient. Treatment decisions were individualized based on patient-specific factors, age (Table 1), comorbidity burden, performance status (not documented in all patients), and consent to treatment. Patients with high comorbidity burden or who were medically frail received local therapy only (intravitreal rituximab/methotrexate ± ocular radiotherapy) to minimize systemic toxicity. Younger patients with good performance status received systemic chemotherapy ± local therapy. Treatment included one or more of the following: intravitreal administration of rituximab and/or methotrexate, ocular radiotherapy, and systemic chemotherapy. Eleven patients received at least one of these treatment modalities (Table 2), while one patient died of a cause unrelated to lymphoma before therapy initiation.

**Table 2:**
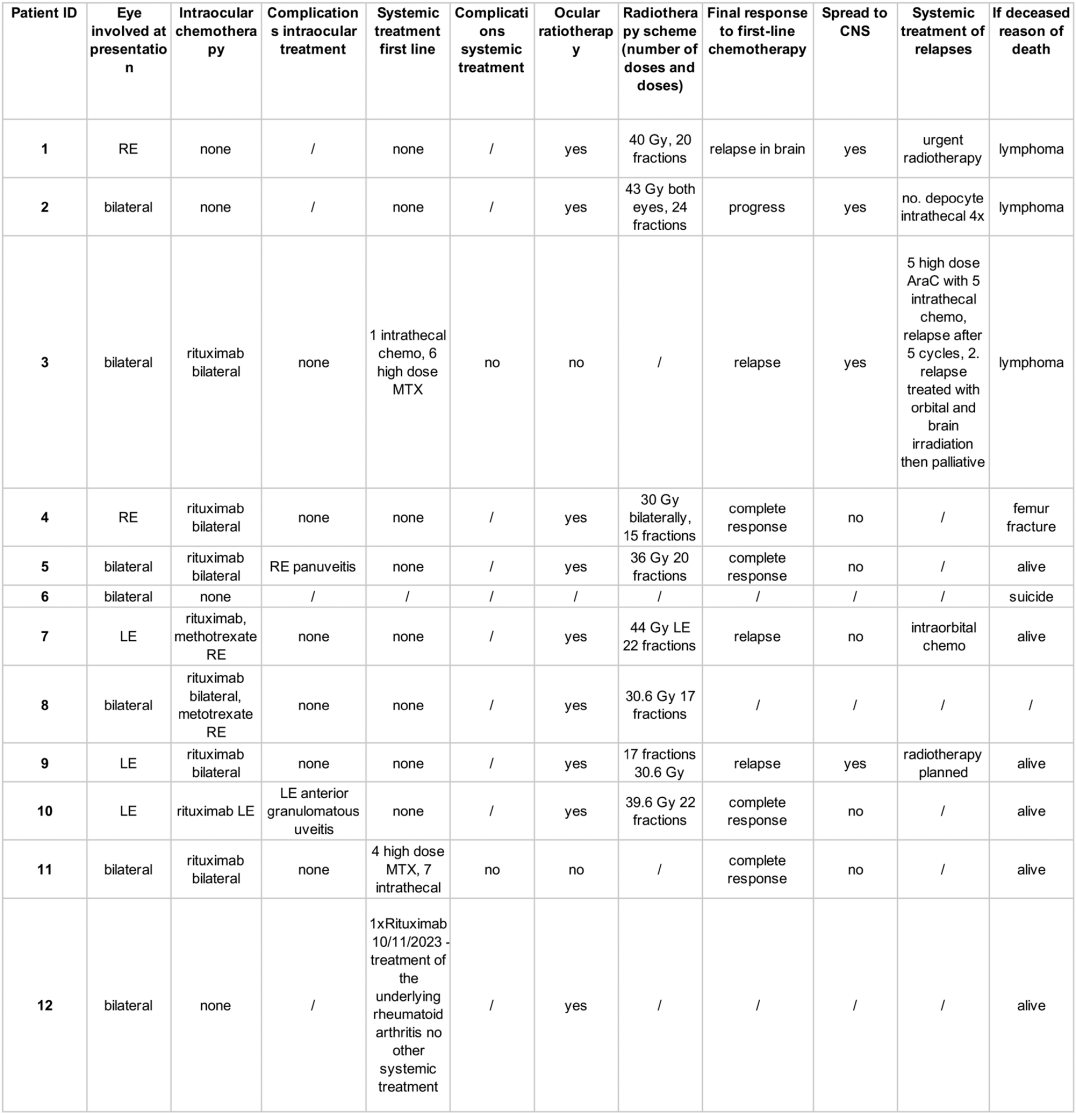
Treatment modalities and outcomes for 12 patients

RE – right eye, LE – left eye, MTX – methotrexate, Gy – Gray, CNS – central nervous system

#### Local treatment outcomes

Local treatment strategies included intravitreal applications of rituximab and/or methotrexate and radiotherapy. All 11 patients who initiated treatment received at least one form of local therapy (Table 2).

#### Intraviteral treatment

Rituximab (1 mg/0.1 mL) was the first-line intravitreal treatment and was administered to eight of 11 patients (14 eyes). The intravitreal treatment protocol was adapted to a multimodal treatment strategy. Among the eight patients, seven had bilateral disease, and one had unilateral disease. In one case of bilateral disease, one eye was not treated with intravitreal rituximab due to the presence of silicone tamponade left in the vitreous space after diagnostic retinal biopsy. In two patients, intravitreal rituximab was discontinued prematurely because of the development of granulomatous uveitis. Three patients did not receive any intravitreal therapy—one patient received systemic rituximab instead—while the reasons for treatment abstention in the other two patients were not available. In addition, two patients also received intravitreal methotrexate (400 mg/mL injections) with no reported complications.

#### Ocular radiotherapy

Patients were treated with upfront radiotherapy when they were not suitable for systemic treatment due to their age, comorbidities, and performance status. Radiotherapy targeted one or both eyes and, in relapse settings, was extended to the whole brain or cerebrospinal axis, according to the recommendations of the tumor board.

Two patients were treated with electron beams, whereas eight patients received photon beams (6 MV energy). Treatment methods included three-dimensional conformal radiation therapy or volumetric modulated arc therapy to deliver at least 98% of the prescribed dose to 95% of the target volume.

The two patients treated with electrons received total doses (TD) of 40 Gy and 43.2 Gy to the right eye and both eyes, respectively, but subsequently experienced disease progression and were subsequently treated with whole-brain irradiation (45 Gy).

The remaining eight patients received photon doses as follows: five received 30 Gy, one received 36 Gy, one received 39.6 Gy, and one received 44 Gy. Six patients were irradiated to both their eyes and orbits, and two were irradiated to the left eye and orbit only. Additionally, four patients underwent whole-brain irradiation following disease progression.

#### Systemic treatment

Among the 12 patients, only two received systemic treatment as frontline therapy for PVRL. One patient, a 66-year-old woman, was treated with high-dose methotrexate (5 g/m²) for six cycles combined with intrathecal chemotherapy (methotrexate, cytosine arabinoside, and dexamethasone). She achieved a complete response but relapsed after ten months. The second patient, a 52-year-old woman, received high-dose methotrexate (8 g/m²), rituximab (375 mg/m²), and intrathecal chemotherapy (methotrexate, cytosine arabinoside, and dexamethasone). She achieved complete remission, which remained ongoing at the last follow-up.

None of the patients underwent high-dose chemotherapy with autologous stem cell transplantation.

### Prognosis and survival

At the first visit to Eye Hospital, UMC Ljubljana, five out of 12 patients presented with unilateral disease, whereas seven patients presented with bilateral disease at onset. Among the patients with unilateral presentation, three patients were diagnosed with contralateral eye involvement: one patient developed contralateral disease 29 days after the initial diagnosis, another after 8 months, and another after 13 months. Two patients presented with unilateral disease at the last follow-up.

**Fig. 2:**
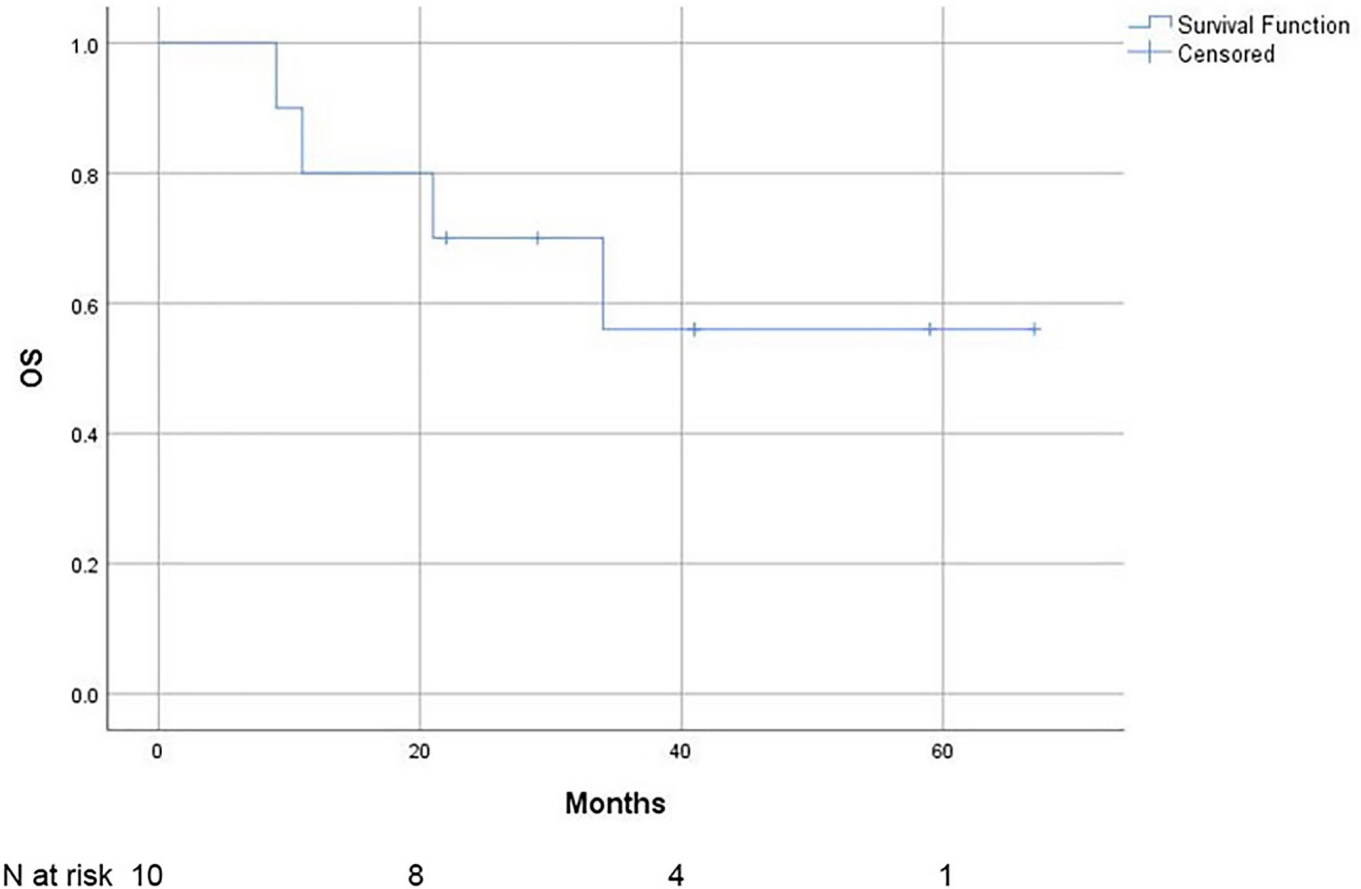
Overall survival (OS) of patients with primary vitreoretinal lymphoma (*N* = 10)

One patient who died by suicide before treatment initiation and one who was lost to follow-up after treatment initiation were excluded from the survival analysis, leaving 10 evaluable patients. During the observation period, 4 out of 10 patients died, one from causes unrelated to lymphoma. The median OS was not reached; the 2-year OS was 70% (95% CI 55.5% − 84.5%), and the 3-year OS was 56% (95% CI 38.9%-73.1%). The OS curve is shown in Fig. 2.

**Fig. 3:**
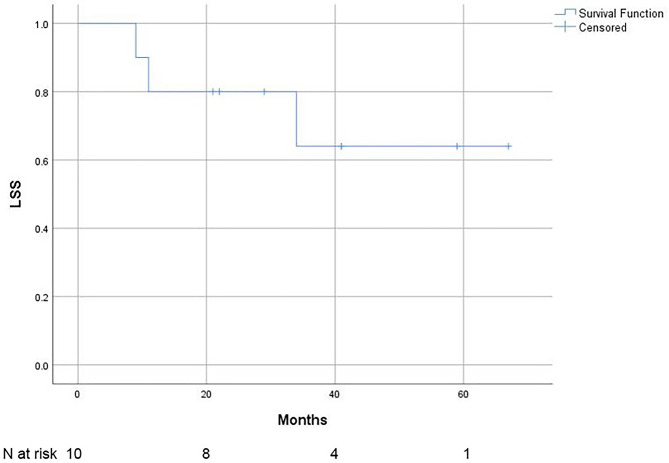
Lymphoma-specific survival (LSS) of patients with primary vitreoretinal lymphoma (*N* = 10)

The 2-year LSS rate was 80% (95% CI 92.6% − 67.4%), and the 3-year LSS rate was 64% (95% CI 81.5% – 46.5%). The LSS is shown in Fig. 3.

**Fig. 4:**
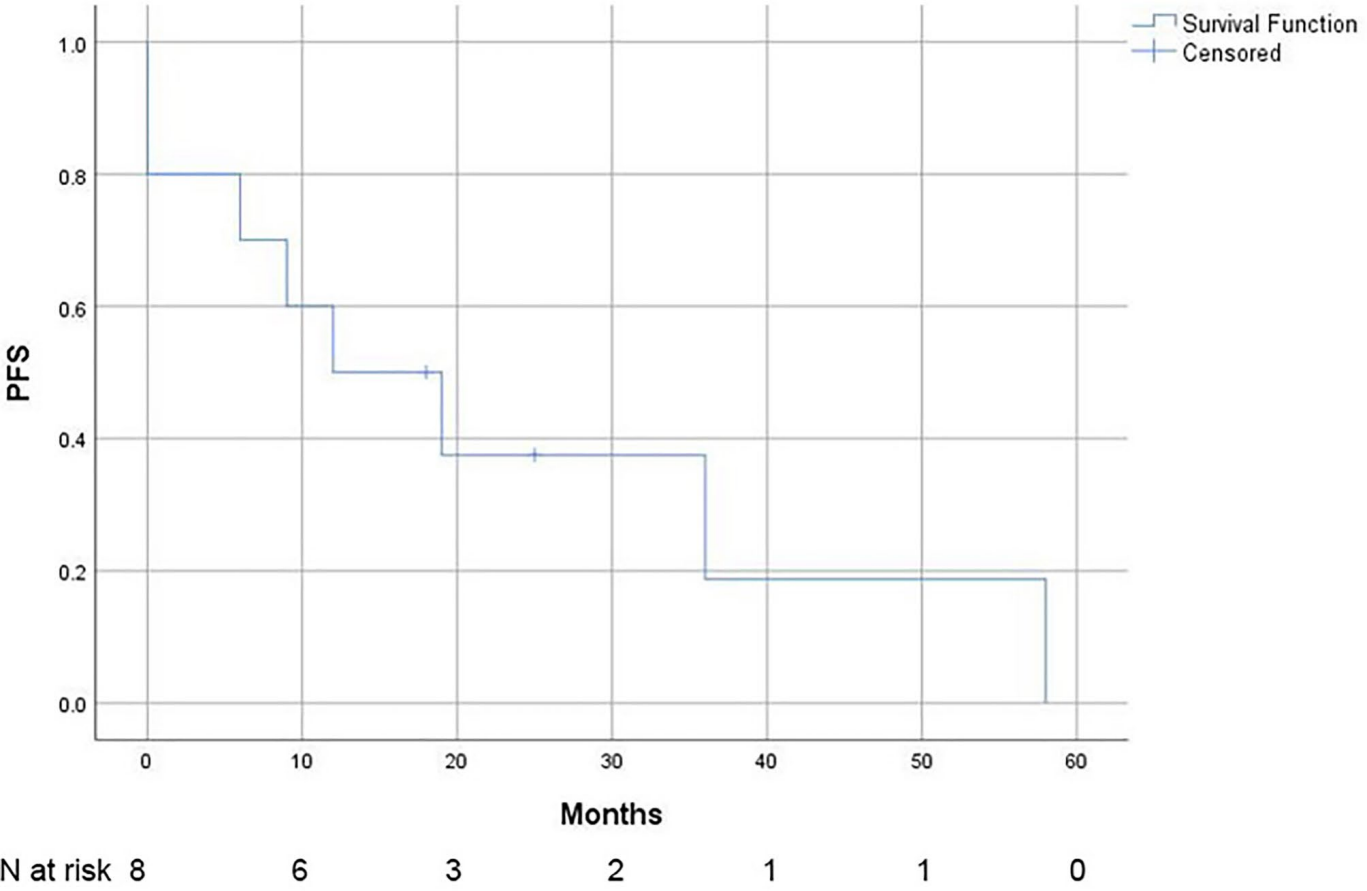
Progression-free survival (PFS) of patients with primary vitreoretinal lymphoma (*N* = 10)

Among the 10 patients, 8 experienced disease progression during the observation period. The median PFS was 12 months (95% CI 0–26 months). The 2-year PFS was 37.5% (95% CI 53.6% − 21.4%), and the 3-year PFS was 18.8% (95% CI 34.3% − 3.3%). The PFS data are presented in Fig. 4.

## Discussion

This population-based study represents the first comprehensive national evaluation of PVRL. By including all patients diagnosed and treated at the sole Slovenian referral center over more than a decade, our findings contribute valuable real-world data on national incidence, clinical characteristics, and treatment strategies with outcomes for this rare and aggressive malignancy.

The study revealed an average yearly incidence of 0.52 cases of PVRL per million in Slovenia, which is consistent with prior reports [2, 14]. The median age at diagnosis was 75 years, with a female predominance, reflecting the typical demographics reported in other studies [15, 16]. Most patients presented with decreased visual acuity (75%) and floaters (58%), which is consistent with published data [17, 18].

Although referral diagnoses to the Eye Hospital were diverse, PVRL often masqueraded as an inflammatory condition. These included uveitis (intermediate, posterior, or panuveitis) and, in one case, nonarteritic anterior ischemic optic neuropathy. The most frequent and consistent sign revealed by our first tertiary in-hospital examination was vitreous cellular infiltration (91.7%), which presented with a uveitis-like appearance, while optic nerve infiltration was uncommon, aligning with published data [4, 19–21]. In our study, detailed retinal data were limited by inconsistent clinical documentation and frequent dense vitreous opacities, limiting fundus visualization, reflecting real-world challenges.

Our study highlights the diagnostic challenges of PVRL, with a median delay of approximately 8 months from symptom onset to definitive diagnosis. This delay parallels international reports of 12–24 months in large review studies and highlights the ongoing global challenge of recognizing PVRL, despite increasing awareness [19]. Diagnostic delays in our study were ultimately overcome through systematic tissue-based sampling. Initial misdiagnoses (e.g., posterior uveitis, retinal vasculitis) stem from PVRL’s mimicry of inflammatory conditions. Our protocol, which combines vitreous biopsy (100% of cases), retinal sampling (42%), and flow cytometry, enhanced diagnostic accuracy, in line with recommendations to integrate cytopathology and immunophenotyping, as cytology alone has a negative predictive value of only 60% [20]. Furthermore, the application of 41Gauge S-FNAB, a novel surgical technique for retinal biopsy, represents a technical advancement. While not yet widely adopted, S-FNAB is a safe and efficient method that provides sufficient and high-quality specimens even from microretinal infiltrates [10].

The study reflects real-world diagnostic practice during 2013–2024, when molecular markers such as MYD88 and cytokine testing were not yet widely established for PVRL. All diagnoses were confirmed through comprehensive cytopathologic assessment and clinical correlation, consistent with contemporary standards at the time of patient enrollment. In our study, PVRL was diagnosed by cytopathological examination in 11 cases and histopathological examination in one case. Cytopathological diagnoses were based on microscopic examination of Giemsa- and Papanicolaou-stained cytospins, multi-color flow cytometric immunophenotyping and/or molecular analyses. The diagnostic approach was stepwise, beginning with microscopic evaluation and flow cytometric analysis, followed by molecular testing. When sufficient DNA was isolated from cytological samples, gene rearrangement studies and/or detection of MYD88 mutations were performed. Molecular testing proved crucial for establishing the diagnosis in cases where flow cytometric results were difficult to interpret. Flow cytometric analysis was performed in all cases and supported the diagnosis of PVRL in 10/12 (83.3%) cases, comparable to previously reported flow cytometry results in the literature, where 82.4% sensitivity and 100% specificity was achieved [22]. Although flow cytometry provides valuable information on cell lineage, clonality and antigenic characteristics of lymphoma cells, crucial for PVRL diagnosis, it is not widely used as diagnostic approach in intraocular lymphoma diagnostics due to the need for expensive equipment and highly trained personnel. Consequently, immunocytochemistry and molecular tests are therefore used instead. In our study, gene rearrangement studies and detection of MYD88 mutation were performed in 7 and 4 cases, respectively. However, gene rearrangement studies failed to confirm lymphoma in two cases, and MYD88 mutation in one case. Notably, in one case with inconclusive flow cytometric results, the diagnosis of PVRL was established by detecting a MYD88 mutation. Our results are consistent with published data, which report 65–95% sensitivity for gene rearrangement studies [17] and 75.9% sensitivity with 100% specificity detecting MYD88 mutation [23]. Since detection of MYD88 mutation is more specific than gene rearrangement studies, we recommend performing it in all cases with inconclusive flow cytometric results or in centers where flow cytometry is not available. In challenging cases, the measurement of interleukin (IL)-10 and IL-6 concentrations in vitreous samples, as well calculation their ratio (IL-10/IL-6) and ISOLD score, might also aid in the diagnostics of PVRL [24].

Owing to older age and comorbidities, our treatment paradigm favored local therapy over systemic treatment with only two of 11 treated patients, receiving frontline systemic therapy. However, the 2024 EHA–ESMO guidelines recommend high-dose methotrexate–based regimens as frontline therapy for fit patients [7]. The majority of patients received ocular radiotherapy (73%) with selective intravitreal treatment. Local treatment achieved ocular remission in all patients, even when it was discontinued due to ocular toxicity. Although specific dose‒response analyses are limited by individualized protocols, patients who completed intravitreal therapy achieved favorable local control. Intravitreal treatment complications were manageable and consistent with published data [25–29]. No complications were reported in two patients receiving methotrexate. Among rituximab-treated eyes, granulomatous uveitis occurred in 14% (2/14 eyes), within the reported range [26].

Ocular radiotherapy was delivered at doses of 30–44 Gy, reflecting contemporary oncologic protocols at the time of treatment [19, 28]. While recent studies advocate reduced doses (23.4 Gy) combined with systemic therapy, our higher doses reflect standard practices for elderly patients who are unfit for systemic treatment. Two patients who underwent electron beam therapy (40–43.2 Gy) subsequently developed CNS progression, highlighting the role of the blood‒brain barrier in compartmentalizing ocular disease and the inherent risk of CNS involvement in PVRL regardless of local treatment intensity.

Treatment heterogeneity in our study reflects individualized real-world decision-making guided by patient-specific factors, age, comorbidity burden, and performance status. Local therapy was prioritized for elderly patients with comorbidities to minimize toxicity while achieving excellent local control. One of the two patients who received initial systemic therapy and seven of the eight patients who received no systemic therapy experienced relapse during follow-up. Notably, both patients treated with ocular radiotherapy alone died of the disease. Ostrovsky et al.‘s comprehensive review of 476 patients across 28 studies revealed no significant difference in overall survival between ocular treatment alone and systemic or combined therapy. A significantly lower risk of relapse was reported in patients receiving ocular or combined therapy than in those receiving systemic therapy alone [30]. Importantly, this meta-analysis was based exclusively on retrospective studies with relatively small sample sizes and wide confidence intervals, limiting the robustness of the conclusions.

While local therapy effectively controlled intraocular disease, four out of ten patients died during follow-up (three lymphoma-related), underscoring PVRL’s systemic lethality despite ocular remission. This highlights the need for integrated oncologic management.

Bruton tyrosine kinase inhibitors (e.g. ibrutinib, acalabrutinib), recommended by EHA-ESMO 2024 guidelines for CNS lymphoma, represent promising oral options for elderly patients. However, BTKi are not routinely available in Slovenia for frontline PVRL treatment due to regulatory and reimbursement restrictions for this specific indication. Future access to these agents could improve systemic control in medically frail patients.

Treatment modality, time from symptom onset to diagnosis, age, and bilateral involvement have been associated with prognosis in prior studies. Owing to our small sample size, these correlations could not be robustly assessed.

In our cohort, the median time from symptom onset to diagnosis was seven months, with no clear correlation observed between diagnostic delay and the risk of progression. This finding aligns with Ostrovsky’s 2024 meta-analysis of 476 patients, which found no correlation between diagnostic delay (≤ 6 vs. > 6 months) and overall or progression-free survival [30].

Bilateral involvement was present in 55% of patients at diagnosis, with three additional patients developing contralateral disease (82% final rate). This high prevalence of bilateral disease is consistent with contemporary literature, where rates of bilateral involvement range from 33.3% to 72% [31]. This high prevalence reinforces the necessity of lifelong bilateral surveillance, as contralateral involvement can occur years after initial presentation [32].

Older age is associated with increased overall mortality, but our cohort was too small to analyze this association. Three deaths were unrelated to lymphoma, highlighting the frailty of our cohort. This observation supports the rationale for individualized treatment approaches, prioritizing quality of life and minimizing toxicity.

The small cohort size (12 patients overall, with 10 included in the survival analysis) represents a major limitation of this study. Although this constraint reflects the rarity of primary vitreoretinal PVRL, the limited sample size reduces the statistical power of the survival analyses and limits the robustness and generalizability of the PFS and OS estimates. Accordingly, these outcomes should be interpreted with care.

As this was a retrospective study with a small sample size, our ability to assess survival differences was limited. However, the population-based design reduces selection bias—an important strength in the study of rare diseases such as PVRL. While definitive conclusions about prognostic factors cannot be drawn, our findings offer valuable real-world insights. Prospective, multicenter studies are needed to refine risk stratification and optimize treatment strategies.

## Conclusions

This Slovenian population-based study offers novel insights into the real-world management of PVRL. Treatment was individualized taking into account age and comorbidities, with only 18% of patients receiving frontline systemic therapy due to medical frailty. Local therapies allowed excellent ocular control (100% remission) with acceptable toxicity, while median OS was not reached (2-year OS: 70%, 3-year OS: 56%).

Key clinical observations highlight the importance of recognizing vitreous cellular infiltration in elderly patients as a potential manifestation of PVRL, warranting prompt specialized evaluation and tissue-based diagnosis. The substantial diagnostic delay (median 238 days) and persistent challenges in PVRL detection emphasize the need for heightened clinical awareness among ophthalmologists.

Treatment approaches favoring local therapy, including ocular radiotherapy and selective intravitreal methotrexate or rituximab, have produced outcomes comparable to those of international cohorts [6] with manageable toxicity profiles.

Clinical decision-making should be individualized, considering guideline recommendations, patient comorbidities, and the limitations of the available evidence. Multiinstitutional prospective studies are urgently needed to clarify the comparative efficacy of systemic versus local therapies in PVRL. Emerging systemic therapies like BTKi (EHA-ESMO 2024) may be effectively used even in elderly patients, but their access remains limited.

## Data Availability

The datasets used and/or analyzed during the current study are available from the corresponding author upon reasonable request.

## Abbreviations

BCVA: Best-corrected visual acuity
CNS: Central nervous system
LP: Lumbar puncture
LSS: Lymphoma-specific survival
MRI: Magnetic resonance imaging
OS: Overall survival
PET: Positron emission tomography
PFS: Progression-free survival
PVRL: Primary vitreoretinal lymphoma
s-FNAB: Silicone fine-needle aspiration biopsy
TD: Total dose
UMC: University medical center
